# Multi-Year Mapping of Major Crop Yields in an Irrigation District from High Spatial and Temporal Resolution Vegetation Index

**DOI:** 10.3390/s18113787

**Published:** 2018-11-06

**Authors:** Bing Yu, Songhao Shang

**Affiliations:** State Key Laboratory of Hydroscience and Engineering, Tsinghua University, Beijing 100084, China; b-yu14@mails.tsinghua.edu.cn

**Keywords:** yield estimation, random forest, HJ-1A/1B, normalized difference vegetation index, Hetao Irrigation District

## Abstract

Crop yield estimation is important for formulating informed regional and national food trade policies. The introduction of remote sensing in agricultural monitoring makes accurate estimation of regional crop yields possible. However, remote sensing images and crop distribution maps with coarse spatial resolution usually cause inaccuracy in yield estimation due to the existence of mixed pixels. This study aimed to estimate the annual yields of maize and sunflower in Hetao Irrigation District in North China using 30 m spatial resolution HJ-1A/1B CCD images and high accuracy multi-year crop distribution maps. The Normalized Difference Vegetation Index (NDVI) time series obtained from HJ-1A/1B CCD images was fitted with an asymmetric logistic curve to calculate daily NDVI and phenological characteristics. Eight random forest (RF) models using different predictors were developed for maize and sunflower yield estimation, respectively, where predictors of each model were a combination of NDVI series and/or phenological characteristics. We calibrated all RF models with measured crop yields at sampling points in two years (2014 and 2015), and validated the RF models with statistical yields of four counties in six years. Results showed that the optimal model for maize yield estimation was the model using NDVI series from the 120th to the 210th day in a year with 10 days’ interval as predictors, while that for sunflower was the model using the combination of three NDVI characteristics, three phenological characteristics, and two curve parameters as predictors. The selected RF models could estimate multi-year regional crop yields accurately, with the average values of root-mean-square error and the relative error of 0.75 t/ha and 6.1% for maize, and 0.40 t/ha and 10.1% for sunflower, respectively. Moreover, the yields of maize and sunflower can be estimated fairly well with NDVI series 50 days before crop harvest, which implicated the possibility of crop yield forecast before harvest.

## 1. Introduction

Food security is one of the major challenges that humanity is facing. The Food and Agriculture Organization (FAO) reported that there were about 815 million people worldwide suffering from food shortages in 2016 [1]. To support food security, monitoring and estimating of crop yields in large areas is of great significance [2]. Moreover, accurate and real-time estimation of major crop yields is helpful for decision makers to formulate informed food trade policies [3,4,5]. Crop yield estimation are often based on official statistics derived from crop yield survey performed at some administrative level that are made available several days or months after crop harvesting [6,7,8]. Therefore, developing a simple and convenient yield estimation model that can estimate crop yield timely in different spatial scales is urgently required.

In recent years, remote sensing has been widely used in agricultural monitoring because of its extensive coverage and regular revisit, which makes regional crop identification [9,10] and yield estimation [6,11] possible at acceptable accuracy. The crop yield estimation models based on remote sensing data mainly include three types, i.e., empirical models, crop growth models, and radiation use efficiency (RUE) models.

The empirical or statistical models are based on the relationship between remote sensing indexes and crop yields in selected spatial units. The most frequently used remote sensing indexes include the Normalized Difference Vegetation Index (NDVI) [12,13,14], the Enhanced Vegetation Index (EVI) [15,16], the Leaf Area Index [17], and the Soil Adjusted Vegetation Index (SAVI) [4]. Most available studies have shown that there is a strong linear relationship between these remote sensing indexes and crop yields [18,19,20]. Although empirical models are simple and easy to use, these models usually consider only a few key indexes and neglect other influence factors, which would affect the accuracy of crop yield estimation.

The crop growth models use indexes derived from remote sensing to simulate crop growth and yield [21,22,23]. The crop growth process and yield can be well simulated based on accurate model inputs, including climate, soil and agricultural management measures [24,25]. However, due to the spatial heterogeneity of field conditions, agricultural management, crop planting dates at regional scale, and the complexity of the land uses, the application of crop growth models was generally limited to small areas [26]. At present, crop growth models are usually driven by field measured data and are difficult to extend to large areas where there is a lack of spatial field measured data [27,28,29].

The RUE models estimate crop yield based on gross primary productivity (GPP) or net primary productivity (NPP) derived from remote sensing data [30]. Compared with the crop growth model, the RUE models need less parameters and has certain crop physiological basis. Main parameters for calculating crop yields include RUE, absorbed photosynthetically active radiation (APAR), and harvest index (HI) [31,32,33]. However, some parameters, especially HI, are difficult to obtain and usually estimated by field experiment or experience. The estimated parameters may neglect the effects of some important environmental factors, which will bring uncertainty to crop yield estimation.

Therefore, there are still some problems in regional crop yields estimation using the existing methods. In recent years, machine learning methods, including random forest (RF), support vector machine (SVM), and artificial neural network (ANN), have been gradually used in crop identification based on remote sensing data [34,35,36]. More recently, these machine learning methods have been applied to crop yield estimation. The ANN has been successfully applied to yield estimation of various crops, such as maize [11], wheat [37], potato [38], melon [39] and grassland dry matter yield [40]. The RF method has also been used in crop yield estimation, especially for large areas of maize [7,41,42], soybean [43] and wheat [44]. Therefore, most available studies were on yield estimations of maize and wheat, but few studied yield estimations of sunflower, an important economic crop in arid regions of Northwest China.

The key factors of crop yield estimation using machine learning methods are the determination of predictors and model calibration and validation. The periodic variation of vegetation index is closely related to crop growth period, and is therefore usually used as predictors [45,46]. Since most crops have growth periods of several months, remote sensing data with fine temporal resolution are usually needed, such as moderate resolution imaging spectroradiometer (MODIS) [7] or advanced very high-resolution radiometer (AVHRR) [6]. However, the finest spatial resolution of these remote sensing data is 250 m, which will affect the feature extraction of crops from possible mixed pixels, especially in small-sized cropland blocks that is common in China [47]. The inaccuracy of model predictors would inevitably lead to uncertainty in crop yield estimation. Therefore, it is necessary to make use remote sensing data with both high temporal and spatial resolutions to improve the yield estimation accuracy. In the processes of model calibration and validation, the precision of crop distribution maps has a great influence on yields extraction of different crops [7]. Therefore, a high precision crop distribution map is the basis for the calibration and validation of crop yield estimation models.

China launched the HJ-1A/1B CCD satellite constellation with 30 m spatial resolution and two days revisit period on 6 September 2008 [48]. Previous studies have indicated that NDVI derived from HJ-1A/1B CCD images could be applied to regional crop identification [49,50] and phenological characteristics estimation [51]. However, few studies had applied HJ-1A/1B CCD data to crop yield estimation in arid and semi-arid areas [52]. Yu and Shang [49] mapped multi-year maize and sunflower in Hetao Irrigation District from 2009 to 2015 with the mean relative statistical error of 10.82% for maize and 4.38% for sunflower, which provided the basis for further yield estimation in this region. The main objective of this study is to develop practicable RF models for yield estimation of major crops in Hetao Irrigation District of China based on the NDVI series derived from HJ-1A/1B CCD images and high precision crop distribution maps.

## 2. Study Area

**Figure 1:**
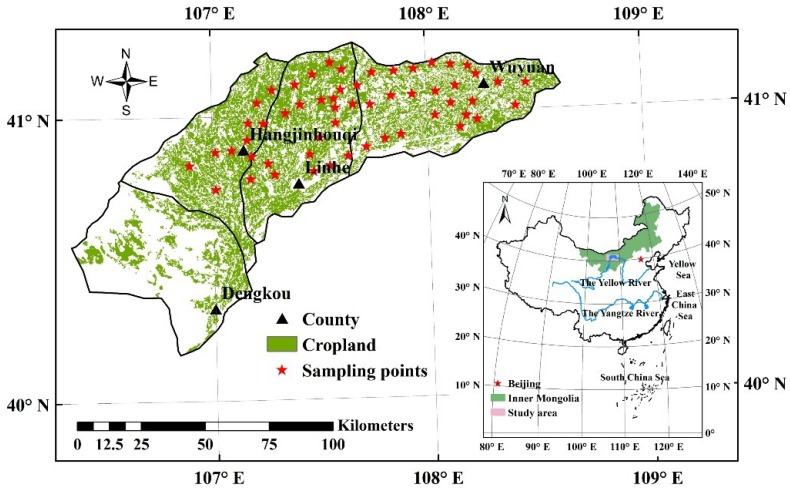
Location and cropland distribution of the study area and sampling points in 2014 and 2015.

Four counties in western and middle Hetao Irrigation District (HID) were selected as the study region, covering an area of 0.91 million ha with 44% being cropland (Figure 1). The HID is the third largest irrigation district and an important food production base in China located in the western part of the Inner Mongolia Autonomous Region in North China. The HID lies in a typical arid area and mainly relies on the Yellow River water for irrigation, while a water-saving rehabilitation program has been carrying out since 1998 to reduce water diversion from the Yellow River and slow down the rise of the groundwater table caused by irrigation [53,54]. Consequently, the decrease of available irrigation water influenced the crop planting structure and crop yield in HID.

The dominant crops include maize, sunflower and wheat, and the crop planting structure has changed significantly in recent years due to economic and irrigation factors. According to the statistics of the crop planting area provided by the local government, the proportion of maize planting area increased from 28% to 40%, sunflower increased from 31% to 40%, and wheat decreased from 22% to 13% from 2010 to 2015. Since maize and sunflower accounted for about 80% of the crop planting area in recent years, only maize and sunflower were considered in this study. According to field investigation in HID, the growth period of maize is generally from the 120th to 260th day in a year, while the sunflower from the 160th to 260th day. Moreover, maize and sunflower both reached their peak growth periods at about the 220th day in the year [49].

## 3. Data and Method

### 3.1. Data Sources

Data used in the present study mainly include HJ-1A/1B CCD images, field measured and official statistical crop yield data, crop distribution and land use maps.

The two-day-repeat CCD sensors of Chinese HJ-1A/1B satellites capture ground features with 30 m pixel resolution, with each satellite carrying a 4-band multispectral optical imagers that captures radiation in the blue (0.43–0.52 μm), green (0.52–0.60 μm), red (0.63–0.69 μm), and near-infrared (0.76–0.90 μm) bands [48]. Level 2A images for HID covering the crop growth period from April to October in the years from 2010 to 2015 with the cloud cover less than 5% were downloaded from the China Centre for Resources Satellite Data and Application (CRESDA) [55].

Crop type and yield were surveyed on the ground in 2014 and 2015. Fifty-five sampling points were first determined considering the spatial distribution uniformity and the homogenous of crop type within the area of cropland based on the 1:100,000 land use map of the cropland in 2005 (Figure 1) provided by National Earth System Science Data Sharing Infrastructure [56]. Distributing sampling points uniformly throughout the entire study area ensured the representativeness and diversity of crops with different growth conditions and crop yield. Then we conducted field survey about crop type and yield based on the location of the sampling points on the map. Each sampling point represented an area of over one hectare with the same crop, which could prevent the possible existence of mixed pixels in developing remote sensing-based models for crop yield estimation. Meanwhile, the crop type of each point may be different in these two years due to crop rotation. As a result, thirty-four sampling points of maize, fifty-four points of sunflower, and twenty-two points of other crops were obtained in 2014 and 2015, and the statistics of measured crop yields at the sampling points were shown in Table 1. The spatial resolution of HJ-1A/1B CCD images was 30 m and the area of each sampling point was over one hectare, then we selected eight pixels (30 × 30 m^2^) at each sampling point. Consequently, we have 272 and 432 pixels in total with measured yield data for model calibration for maize and sunflower, respectively. For each sampling point, we selected thirty plants with relatively uniform growth to measure the average crop yield. The sampled maize cob and sunflower plate were air-dried, and the maize and sunflower seeds were threshed and weighed to obtain the dry grain weight. Then, the crop yields per unit area of the sampling point were calculated considering the crop density (Table 1).

For the selected four counties in HID, we obtained the average maize and sunflower yield statistics per county from 2010 to 2015 from the Bayannur Agricultural Information Network [57]. We also used the crop distribution maps with 30 m spatial resolution of maize and sunflower from 2010 to 2015 obtained in our previous study [49].

### 3.2. Data Processing and Determination of Model Input

The preprocessing procedures for HJ-1A/1B CCD images mainly include radiometric calibration and atmospheric and geometric corrections [49]. In case of HJ-1A/1B CCD images, the band 4 (near-infrared band) and the band 3 (red band) were used to calculate NDVI [58].

(1) $$\mathrm{NDVI}=\frac{\left(\rho_{t,4}-\rho_{t,3}\right)}{\left(\rho_{t,4}+\rho_{t,3}\right)}\;$$

**Figure 2:**
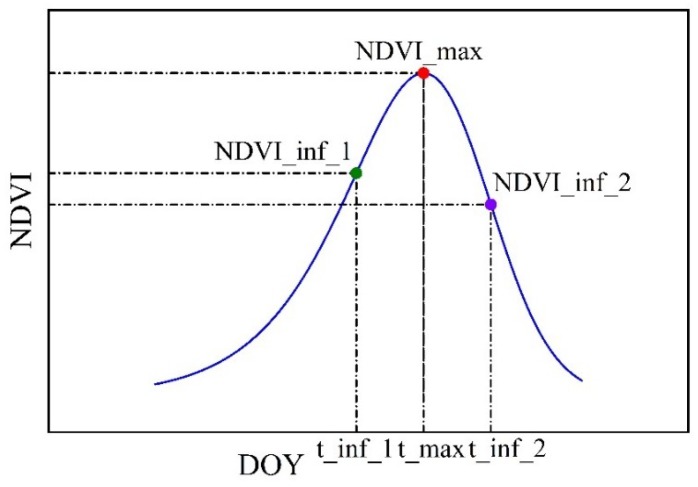
Sketch of asymmetric logistic curve to fit the NDVI series and characteristic points.

To get daily NDVI series, we used an asymmetric logistic curve (Figure 2) [59] to fit the NDVI series of the available days, which was then used to calculate the daily NDVI values and extract the crop phenological characteristics [49]. The fitting curve equation is

(2) $$\mathrm{NDVI}=a+(b/k)\cdot {\left(1+n\right)}^{-(k+1)/k}\cdot n\cdot {\left(k+1\right)}^{(k+1)/k}\;$$

(3) n=exp[(t+d⋅ln(k)−c)/d] 

where *t* is day of year (DOY); *a*, *b*, *c*, *d* and *k* are curve parameters to be estimated from available NDVI series by the least-squares method.

After curve fitting, three characteristic points in the fitted NDVI curve (Figure 2) were used to determine the crop phenological characteristics and corresponding NDVI values (Table 2). The equations for calculating the phenological characteristics could be found in Yu and Shang [49].

Eight models were developed for the crop yield estimation with different predictors, each being a combination of NDVI series with a specified time interval and/or phenological characteristics (Table 3). Available studies showed that crop yield is closely related with NDVI [8,13,60], and Shao et al. [7] used MODIS NDVI 16-day composite data to achieve an accurate estimation of maize yield in the United States. Moreover, Bose et al. [37] estimated winter wheat yield accurately using MODIS NDVI data in Shandong province, China. Therefore, to compare the impact of time intervals on yield estimation precision, NDVI series in the crop growth period with time intervals of 5 days and 10 days were selected as the inputs for models 1 and 2, respectively. Considering that the phenological characteristics of crops also have close correlations with crop growth and yield, three phenological characteristics in Table 2 were added to the inputs of model 2 to obtain inputs for model 3. Since parameters *d* and *k* in the fitted NDVI curve also affect the crop growth period, *d* and *k* were added to the inputs of model 3 and used as the inputs for model 4. To test whether crop yield could be accurately estimated using the NDVI series before harvest, the NDVI series before the 210th day (50 days before the harvest) instead of the entire growth period were used as the inputs for model 5. The phenological characteristic in the early growth period, t_inf_1, was added to the inputs of model 5 and used as the inputs for model 6. To further test if these three phenological characteristics and corresponding NDVI indexes in Table 3 can be substitutes of the NDVI series, these six indexes were used as the inputs for model 7. Combination of the inputs of model 7 and parameters *d* and *k* constitutes the input for model 8.

### 3.3. Random Forest Regression Algorithm

The random forest (RF) algorithm is one of the most widely used machine learning methods, which has the advantages of less input parameters, simple operation, and strong stability. It is an ensemble learning method for classification, regression and other tasks. To achieve these tasks, a multitude of decision trees are randomly generated at the training stage, and each decision tree inside would make a decision about the problem independently by selecting a random set of input data [61]. In the case of classification problems, the result depends on the results of most decision trees. While in the case of regression problems, the result depends on the average value of the result of each decision tree [61,62]. In this study, we used RF regression function implemented in the “Random Forest” package within Matlab R2017b software developed by MathWorks (Natick, MA, USA) to estimate the crop yield. Using remote sensing data as inputs, RF regression algorithm has been successfully applied to crop yield estimation in recent years [7,41,42].

The yield estimation in this study is based on pixel-level, and the annual crop yield is estimated from [7]:

(4) $$Y_{i,j,k}=F(x_{i,j,k})\;$$

where $Y_{i,j,k}$ is crop yield at pixel (*i*, *j*) in the *k*th year, *x* is the vector of predictors and *F* is the predictive function of RF regression algorithm.

In the RF regression algorithm, the performance of the model could be improved by adjusting three major parameters. The first one was ntree that represents the number of decision trees with the default value of five hundred, the second one was mtry that represents the number of features used at each node with the default value to be 1/3 of the total number of the features, and the third one is nodesize that represents the minimum size of the terminal nodes of decision trees with the default value of one [63]. Moreover, some studies have shown that the prediction accuracy of the RF model is acceptable when nodesize takes the default value [44,62].

### 3.4. Model Calibration and Validation

We used the field measured crop yield data for the RF model calibration. In previous studies, the calibration of yield estimation models was mostly based on the regional level [7,19]. Here we attempted to calibrate the RF model using yield data at pixel scale to improve the model accuracy. We used the root-mean-square error (RMSE), the relative error (RE), the coefficient of determination (*R*^2^) and the adjusted *R*^2^ (${\bar{R}}^{2}$) to evaluate the performance of RF model driven by different predictors, which can be calculated from

(5) $$\mathrm{RMSE}=\sqrt{\frac{1}{N}\sum_{i=1}^{N}{\left(S_{i}-P_{i}\right)}^{2}}\;$$

(6) $$\mathrm{RE}=\frac{1}{N}\sum_{i=1}^{N}\frac{\left|P_{i}-S_{i}\right|}{P_{i}}\times 100\%\;$$

(7) $$R^{2}=\frac{{\left[\sum_{i=1}^{N}\left(S_{i}-\bar{S})(P_{i}-\bar{P}\right)\right]}^{2}}{\sum_{i=1}^{N}{\left(S_{i}-\bar{S}\right)}^{2}\sum_{i=1}^{N}{\left(P_{i}-\bar{P}\right)}^{2}}\;$$

(8) $${\bar{R}}^{2}=1-(1-R^{2})\frac{N-1}{N-p-1}\;$$

where *S_i_* and *P_i_* (*i* = 1, 2, …, *N*) are the *i* th field measured or official statistical crop production and model estimated crop yields, respectively, $\bar{S}$ and $\bar{P}$ are the corresponding mean values, and *N* is the total number of data used for the RF model calibration or validation, and *p* is the number of predictors of each model.

Among these indexes, ${\bar{R}}^{2}$ was used to adjust *R*^2^ in model calibration to avoid over fitting of the model by considering the influence of predictor numbers. In model validation using statistical crop yield or production, *R*^2^ and ${\bar{R}}^{2}$ was used to indicate the linear correlation of estimated and statistical production, where *p* = 1 is used to adjust *R*^2^ the for the univariate regression analysis. The closer the RMSE and RE to zero and the *R*^2^ and ${\bar{R}}^{2}$ to one, the higher the accuracy of the model.

## 4. Results and Discussion

### 4.1. Model Calibration with Field Measured Yields at Pixel Level

**Figure 3:**
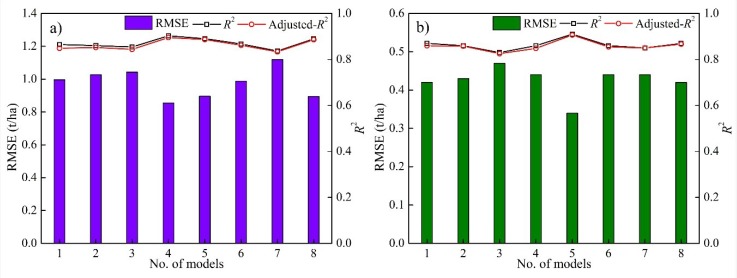
The RMSE (t/ha), coefficient of determination (*R*^2^), and adjusted *R*^2^ of RF models driven by different predictors for maize (**a**) and sunflower (**b**).

We used the measured yields to calibrate the maize and sunflower RF models driven by different predictors, respectively. The calibration performance of RF models was shown in Figure 3. Compared with previous model calibration at county scale [7,11,41], the model calibration in this study was based on pixel scale, which could improve the performance of crop yield estimation model at finer resolution. No matter for maize or sunflower, the *R*^2^ and adjusted *R*^2^ of all RF models were between 0.80 and 0.90, and the difference between *R*^2^ and adjusted *R*^2^ was very small due to the number of calibration data (272 for maize and 432 for sunflower) was far more than the number of predictors, which indicated that there was no over fitting phenomenon in our calibrated models. The RMSE of maize ranged from 0.85 to 1.12 t/ha, while the RMSE of sunflower were lower than the maize and ranged from 0.34 to 0.47 t/ha. For maize, models 1, 2, 4, 5 and 8 had relatively lower RMSE and higher *R*^2^ than the other three models. While for sunflower, models 1, 2, 5, 6 and 8 had relatively lower RMSE and higher *R*^2^ than the other three models.

### 4.2. Model Validation with Statistical Yield and Production at the County Level

We used the above-calibrated RF models to estimate the yields of maize and sunflower, and then compared the estimated yield and production with the official statistical data at the county level in the irrigation district in 6 years (from 2010 to 2015) to further validate the model.

**Figure 4:**
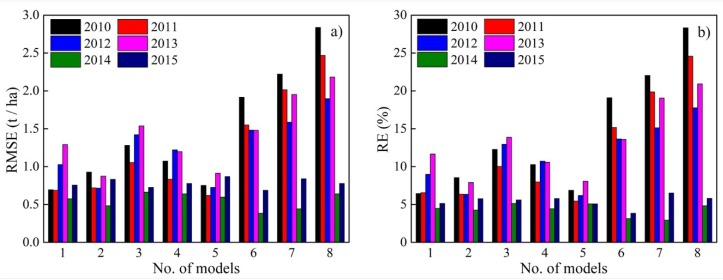
The RMSE (**a**) and RE (**b**) of RF models for maize yield estimation from 2010 to 2015.

**Figure 5:**
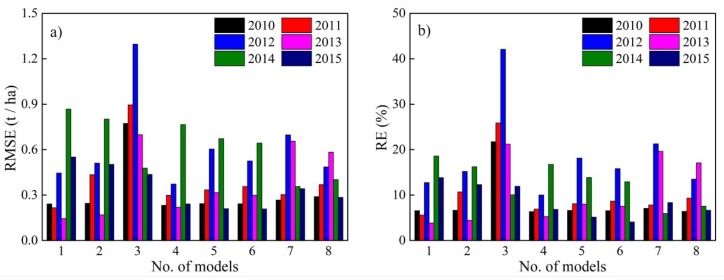
The RMSE (**a**) and RE (**b**) of RF models for sunflower yield estimation from 2010 to 2015.

Figure 4 and Figure 5 showed the inter-annual variations of the accuracy of RF models driven by different predictors for maize and sunflower in the irrigation district, respectively.

For maize, models 1, 2 and 5 had higher estimation accuracy in different years, with the RMSE ranging from 0.57 to 1.29 t/ha, from 0.48 to 0.93 t/ha, and from 0.69 to 0.91 t/ha, and the RE ranging from 4.5% to 11.7%, from 4.3% to 8.5%, and from 5.1% to 8.1%, respectively. These three models also performed better in model calibration. Among these 3 models, model 5 has the highest accuracy with the multi-year average values of RMSE and RE of 0.75 t/ha and 6.1%, respectively.

For sunflower, models 5, 6 and 8 had higher estimation accuracy in different years, with the ranges of RMSE of 0.22–0.77, 0.21–0.64, and 0.28–0.58 t/ha, and corresponding RE of 5.3–16.8%, 4.1–15.8%, and 6.4–17.1%, respectively. Among these models, model 8 has the highest accuracy with the multi-year average values of RMSE and RE of 0.40 t/ha and 10.1%, respectively.

As a result, models 1, 2 and 5 were chosen as the three better models for maize, while models 5, 6 and 8 as the better models for sunflower. The model 5 was among the three better models for both maize and sunflower, which indicates that the yields of maize and sunflower can be estimated fairly well with NDVI series 50 days before crop harvest and implicates the possibility of crop yield forecast before harvest. If we could estimate the NDVI series 50 days before crop harvest without NDVI data of the final growth period using appropriate interpolation or curve fitting methods, then the crop yield can be forecasted before crop harvest. The possibility of crop yield forecast before harvest will be considered in further studies.

Furthermore, we calculated the total production of maize and sunflower in four counties (Dengkou, Linhe, Hangjinhouqi and Wuyuan) using the above eight models by adding up the total production of all maize or sunflower pixels in each county. As a result, we obtained 24 (4 counties in 6 years) estimated total production for maize and sunflower, respectively, which were compared with the official statistical productions during 2010 to 2015 (Table 4). From Table 4, the *R*^2^ and the adjusted *R*^2^ were mostly over 0.45 for maize and over 0.60 for sunflower. The RE were mostly less than 30% for maize and 35% for sunflower. It can also be found that *R*^2^ and the adjusted *R*^2^ for model validation using total production are smaller than that for model calibration using crop yield per unit area, which is reasonable because errors in the estimated total production include errors in both yield estimation and crop classification. Compared with previous crop yield estimation studies using machine learning methods [7,11,43] with the *R*^2^ ranging from 0.2 to 0.8, the accuracy of the present crop yield estimations was acceptable.

**Figure 6:**
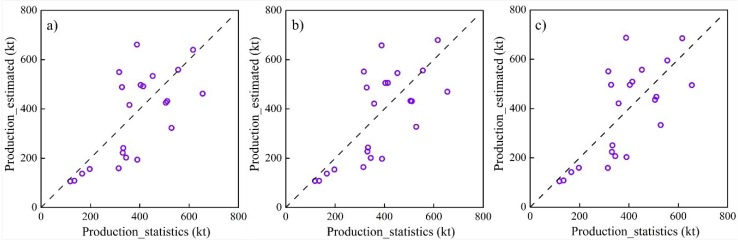
Comparisons of statistical and estimated maize production at the county level using models 1 (**a**), 2 (**b**) and 5 (**c**).

**Figure 7:**
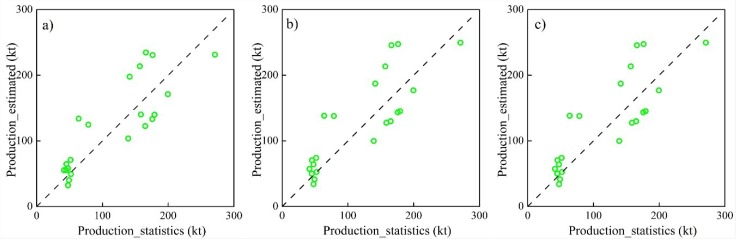
Comparisons of statistical and estimated sunflower production at the county level using models 5 (**a**), 6 (**b**) and 8 (**c**).

The scatter plots for the three better model estimated and statistical productions in four counties during 2010 to 2015 are shown in Figure 6 and Figure 7. For maize, the model 5 has close *R*^2^ values to the other two models and the smallest RMSE and RE values. For sunflower, the model 8 has close RMSE and RE values to the other two models and the highest *R*^2^ value. Consequently, we selected the model 5 to estimate the maize yields, and the model 8 to estimate the sunflower yields.

From the above results, we found that the optimal yield estimation models for maize and sunflower were different. However, there were many studies on the yield estimation of maize [11,19,42,64], a food crop, and few studies on sunflower, an economic crop. Sunflower was an important economic crop in HID because of its obvious drought-tolerance characteristics. This study was the first to develop a regional sunflower yield estimation model, which was of great significance to the management of sunflower planting in HID. Moreover, there were many studies on estimating maize yield in areas where precipitation and temperature were the dominant factors for maize yield while irrigation was not a major influencing factor [7,11,42,64]. Based on the growth conditions of maize in arid areas, the phenological characteristics were added to the yield estimation model in this study, and the yield estimation model suitable for maize in arid area was obtained.

### 4.3. Spatial and Temporal Distribution of Crop Yields

**Figure 8:**
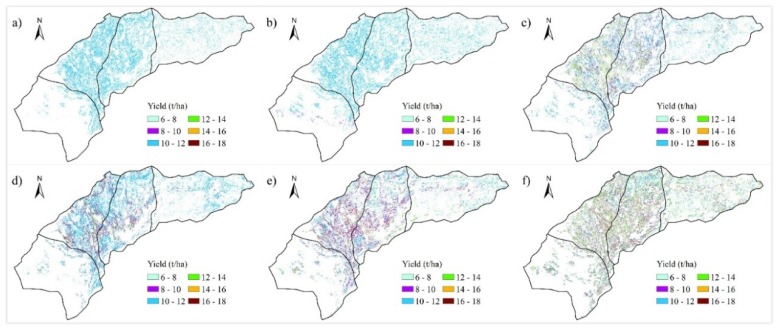
Maize yield maps from 2010 to 2015 (**a**–**f**).

**Figure 9:**
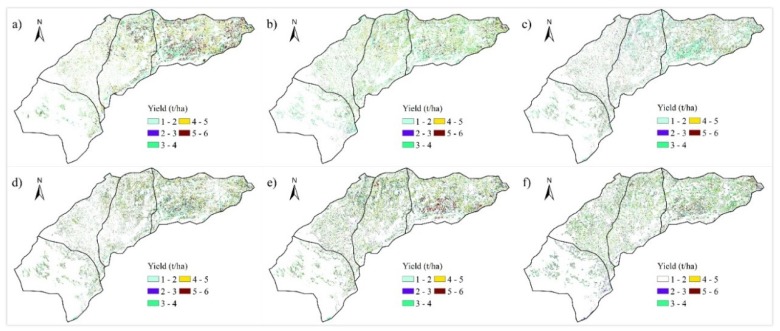
Sunflower yield maps from 2010 to 2015 (**a**–**f**).

Figure 8 and Figure 9 show the yield distribution of maize and sunflower from 2010 to 2015. For maize, most yields fell in the range of 9.23 (the 10th percentile) to 13.43 t/ha (the 90th percentile). The maximum maize yield occurred in Hangjinhouqi, which averaged to 11.08 t/ha, while the minimum yield occurred in Dengkou and Wuyuan, which averaged to 10.83 t/ha. Annual average yields from 2010 to 2015 ranged from 10.62 t/ha in 2011 to 11.41 t/ha in 2015. For sunflower, most yields fell in the range of 2.43 (the 10th percentile) to 5.35 t/ha (the 90th percentile). The maximum sunflower yield occurred in Wuyuan, which averaged to 3.76 t/ha, while the minimum yield occurred in Dengkou, which averaged to 3.64 t/ha. Annual average yields from 2010 to 2015 ranged from 3.54 t/ha in 2012 to 3.87 t/ha in 2014.

The yields of maize and sunflower in Dengkou were both the lowest compared with the other three counties. Possible reason for this spatial variation was that there were more lands with sandy soils in Dengkou, which was not conducive to the growth of maize and sunflower. Meanwhile, maize and sunflower both reached their highest yields in their most distributed counties, Hangjinhouqi and Wuyuan, respectively. The more planted percentage of a crop in an area, the more applicable of the crop in the area. In other words, the present maize and sunflower distributions are generally in agreement with the environment adaptability of these two crops.

## 5. Conclusions

In this study, we used the RF algorithm to estimate annual maize and sunflower yields in Hetao Irrigation District from 2010 to 2015 based on vegetation indexes and phenological characteristics. The main feature of this study was that the regional crop yield estimation of pixel scale was carried out for the first time in HID and this method can be easily applied to other regions, if there were auxiliary crop planting structure map and field measured crop yield data. Main conclusions of this study are as follows:

(1) The RF model could accurately estimate annual regional crop yields, with the multi-year average values of root-mean-square error and the relative error of 0.75 t/ha and 6.1% for maize, and 0.40 t/ha and 10.1% for sunflower, respectively.
(2) Among eight models, the optimal model for maize was NDVI series from the 120th day to the 210th day with 10 days’ interval, while the optimal model for sunflower was the combination of NDVI indexes and phenological characteristics.
(3) The yields of maize and sunflower could be estimated fairly well with NDVI series 50 days before crop harvest, which implicated the possibility of crop yield forecast before harvest.

## Figures and Tables

**Table 1:** Statistics of crop yields at sampling points in Figure 1 in 2014 and 2015.

Statistics	Maize			Sunflower		
	Yield (2014) t/ha	Yield (2015) t/ha	Density plants/ha	Yield (2014) t/ha	Yield (2015) t/ha	Density plants/ha
Minimum	6.225	6.880	33,317	2.772	1.264	19,810
Maximum	14.756	15.627	85,543	5.637	4.299	56,028
Mean	11.092	11.938	61,131	4.190	3.083	33,317
Standard Deviation	2.449	2.057	11,906	0.937	0.970	6003

**Table 2:** Selected phenological characteristics and corresponding NDVI indexes.

No.	NDVI Indexes	Phenological Characteristics
1	NDVI_inf_1, NDVI value of the left inflection point of the NDVI curve with maximum growth rate	t_inf_1, time corresponding to NDVI_inf_1
2	NDVI_inf_2, NDVI value of the right inflection point of the NDVI curve with maximum withering rate	t_inf_2, time corresponding to NDVI_inf_2
3	NDVI_max, maximum value of NDVI curve	t_max, time corresponding to NDVI_max

**Table 3:** Inputs for the crop estimation models.

No.	Model 1	Model 2	Model 3	Model 4	Model 5	Model 6	Model 7	Model 8
1	N_120	N_120	N_120	N_120	N_120	N_120	N_inf_1	N_inf_1
2	N_125	N_130	N_130	N_130	N_130	N_130	N_inf_2	N_inf_2
3	N_130	N_140	N_140	N_140	N_140	N_140	N_max	N_max
4	N_135	N_150	N_150	N_150	N_150	N_150	t_inf_1	t_inf_1
5	N_140	N_160	N_160	N_160	N_160	N_160	t_inf_2	t_inf_2
6	N_145	N_170	N_170	N_170	N_170	N_170	t_max	t_max
7	N_150	N_180	N_180	N_180	N_180	N_180		d
8	N_155	N_190	N_190	N_190	N_190	N_190		k
9	N_160	N_200	N_200	N_200	N_200	N_200		
10	N_165	N_210	N_210	N_210	N_210	N_210		
11	N_170	N_220	N_220	N_220		t_inf_1		
12	N_175	N_230	N_230	N_230				
13	N_180	N_240	N_240	N_240				
14	N_185	N_250	N_250	N_250				
15	N_190	N_260	N_260	N_260				
16	N_195		t_inf_1	t_inf_1				
17	N_200		t_inf_2	t_inf_2				
18	N_205		t_max	t_max				
19	N_210			d				
20	N_215			k				
21	N_220							
…	…							
29	N_260							

Note: N stands for NDVI, and inputs for models 1 to 6 for sunflower start from N_160.

**Table 4:** The accuracies of maize and sunflower production estimations.

Model	Maize				Sunflower			
	RMSE (kt)	RE (%)	*R* ^2^	Adjusted *R*^2^	RMSE (kt)	RE (%)	*R* ^2^	Adjusted *R*^2^
Model 1	131.2	29.2	0.48	0.45	53.1	30.7	0.61	0.59
Model 2	131.0	29.5	0.49	0.47	52.7	31.1	0.61	0.59
Model 3	133.1	29.4	0.51	0.48	54.5	37.8	0.64	0.62
Model 4	132.5	29.6	0.50	0.47	49.6	31.1	0.65	0.64
Model 5	132.0	29.7	0.50	0.48	48.9	32.2	0.66	0.64
Model 6	143.5	30.3	0.44	0.41	47.9	31.7	0.67	0.66
Model 7	153.7	31.7	0.43	0.40	48.0	35.1	0.68	0.66
Model 8	156.9	31.9	0.44	0.41	47.8	33.5	0.68	0.66

## References

[1] The State of Food Security and Nutrition in the World (SOFI) Report. https://www.wfp.org/content/2017-state-food-security-and-nutrition-world-sofi-report.

[2] Hutchinson C.F. (1991). Uses of satellite data for famine early warning in sub-Saharan Africa. Int. J. Remote Sens..

[3] Kowalik W., Dabrowska Z.K., Meroni M., Raczka T.U., de Wit A. (2014). Yield estimation using SPOT-VEGETATION products: A case study of wheat in European countries. Int. J. Appl. Earth Obs. Geoinf..

[4] Noureldin N.A., Aboelghar M.A., Saudy H.S., Ali A.M. (2013). Rice yield forecasting models using satellite imagery in Egypt. Egypt. J. Remote Sens. Space Sci..

[5] Cunha M., Marçal A.R.S., Silva L. (2010). Very early prediction of wine yield based on satellite data from VEGETATION. Int. J. Remote Sens..

[6] Zhang X., Zhang Q. (2016). Monitoring interannual variation in global crop yield using long-term AVHRR and MODIS observations. ISPRS J. Photogramm. Remote Sens..

[7] Shao Y., Campbell J.B., Taff G.N., Zheng B. (2015). An analysis of cropland mask choice and ancillary data for annual corn yield forecasting using MODIS data. Int. J. Appl. Earth Obs. Geoinf..

[8] Johnson D.M. (2014). An assessment of pre- and within-season remotely sensed variables for forecasting corn and soybean yields in the United States. Remote Sens. Environ..

[9] Skakun S., Franch B., Vermote E., Roger J., Becker R.I., Justice C., Kussul N. (2017). Early season large-area winter crop mapping using MODIS NDVI data, growing degree days information and a Gaussian mixture model. Remote Sens. Environ..

[10] Zhong L., Hu L., Yu L., Gong P., Biging G.S. (2016). Automated mapping of soybean and corn using phenology. ISPRS J. Photogramm. Remote Sens..

[11] Fieuzal R., Marais S.C., Baup F. (2017). Estimation of corn yield using multi-temporal optical and radar satellite data and artificial neural networks. Int. J. Appl. Earth Obs. Geoinf..

[12] Mulianga B., Bégué A., Simoes M., Todoroff P. (2013). Forecasting regional sugarcane yield based on time integral and spatial aggregation of MODIS NDVI. Remote Sens..

[13] Son N.T., Chen C.F., Chen C.R., Minh V.Q., Trung N.H. (2014). A comparative analysis of multitemporal MODIS EVI and NDVI data for large-scale rice yield estimation. Agric. For. Meteorol..

[14] Balaghi R., Tychon B., Eerens H., Jlibene M. (2008). Empirical regression models using NDVI, rainfall and temperature data for the early prediction of wheat grain yields in Morocco. Int. J. Appl. Earth Obs. Geoinf..

[15] Bolton D.K., Friedl M.A. (2013). Forecasting crop yield using remotely sensed vegetation indices and crop phenology metrics. Agric. For. Meteorol..

[16] Johnson M.D., Hsieh W.W., Cannon A.J., Davidson A., Bédard F. (2016). Crop yield forecasting on the Canadian Prairies by remotely sensed vegetation indices and machine learning methods. Agric. For. Meteorol..

[17] Fernandez-Ordoñez Y.M., Soria-Ruiz J. (2017). Maize crop yield estimation with remote sensing and empirical models. Proceedings of the IEEE International Geoscience and Remote Sensing Symposium.

[18] Kuri F., Murwira A., Murwira K.S., Masocha M. (2014). Predicting maize yield in Zimbabwe using dry dekads derived from remotely sensed Vegetation Condition Index. Int. J. Appl. Earth Obs. Geoinf..

[19] Ban H., Kim K., Park N., Lee B. (2017). Using MODIS Data to Predict Regional Corn Yields. Remote Sens..

[20] Holzman M.E., Rivas R., Piccolo M.C. (2014). Estimating soil moisture and the relationship with crop yield using surface temperature and vegetation index. Int. J. Appl. Earth Obs. Geoinf..

[21] De Wit A., Duveiller G., Defourny P. (2012). Estimating regional winter wheat yield with WOFOST through the assimilation of green area index retrieved from MODIS observations. Agric. For. Meteorol..

[22] Jin X., Li Z., Yang G., Yang H., Feng H., Xu X., Wang J., Li X., Luo J. (2017). Winter wheat yield estimation based on multi-source medium resolution optical and radar imaging data and the AquaCrop model using the particle swarm optimization algorithm. ISPRS J. Photogramm Remote Sens..

[23] Xie Y., Wang P., Bai X., Khan J., Zhang S., Li L., Wang L. (2017). Assimilation of the leaf area index and vegetation temperature condition index for winter wheat yield estimation using Landsat imagery and the CERES-Wheat model. Agric. For. Meteorol..

[24] Huang J., Ma H., Su W., Zhang X., Huang Y., Fan J., Wu W. (2015). Jointly assimilating MODIS LAI and ET products into the SWAP model for winter wheat yield estimation. IEEE J. Sel. Top. Appl. Earth Obs. Remote Sens..

[25] Cheng Z., Meng J., Qiao Y., Wang Y., Dong W., Han Y. (2018). Preliminary study of soil available nutrient simulation using a modified WOFOST model and time-series remote sensing observations. Remote Sens..

[26] Silvestro P., Pignatti S., Pascucci S., Yang H., Li Z., Yang G., Huang W., Casa R. (2017). Estimating Wheat Yield in China at the Field and District Scale from the Assimilation of Satellite Data into the Aquacrop and Simple Algorithm for Yield (SAFY) Models. Remote Sens..

[27] Zhao Y., Chen S., Shen S. (2013). Assimilating remote sensing information with crop model using Ensemble Kalman Filter for improving LAI monitoring and yield estimation. Ecol. Model..

[28] Ma G., Huang J., Wu W., Fan J., Zou J., Wu S. (2013). Assimilation of MODIS-LAI into the WOFOST model for forecasting regional winter wheat yield. Math. Comput. Model..

[29] Ma Y.P., Wang S.L., Zhang L., Hou Y.Y., Zhuang L.W., He Y.B., Wang F.T. (2008). Monitoring winter wheat growth in North China by combining a crop model and remote sensing data. Int. J. Appl. Earth Obs. Geoinf..

[30] Peng D., Huang J., Li C., Liu L., Huang W., Wang F., Yang X. (2014). Modelling paddy rice yield using MODIS data. Agric. For. Meteorol..

[31] Bandaru V., West T.O., Ricciuto D.M., César I.R. (2013). Estimating crop net primary production using national inventory data and MODIS-derived parameters. ISPRS J. Photogramm. Remote Sens..

[32] Lobell D.B., Asner G.P., Ortiz-Monasterio J.I., Benning T.L. (2003). Remote sensing of regional crop production in the Yaqui Valley, Mexico: Estimates and uncertainties. Agric. Ecosyst. Environ..

[33] Xin Q., Gong P., Yu C., Yu L., Broich M., Suyker A., Myneni R. (2013). A production efficiency model-based method for satellite estimates of corn and soybean yields in the Midwestern US. Remote Sens..

[34] Shao Y., Lunetta R.S. (2012). Comparison of support vector machine, neural network, and CART algorithms for the land-cover classification using limited training data points. ISPRS J. Photogramm. Remote Sens..

[35] Shao Y., Taff G.N., Ren J., Campbell J.B. (2016). Characterizing major agricultural land change trends in the Western Corn Belt. ISPRS J. Photogramm. Remote Sens..

[36] Ng W., Meroni M., Immitzer M., Böck S., Leonardi U., Rembold F., Gadain H., Atzberger C. (2016). Mapping Prosopis spp. with Landsat 8 data in arid environments: Evaluating effectiveness of different methods and temporal imagery selection for Hargeisa, Somaliland. Int. J. Appl. Earth Obs. Geoinf..

[37] Bose P., Kasabov N.K., Bruzzone L., Hartono R.N. (2016). Spiking Neural Networks for Crop Yield Estimation Based on Spatiotemporal Analysis of Image Time Series. IEEE Trans. Geosci. Remote Sens..

[38] Fortin J.G., Anctil F., Parent L., Bolinder M.A. (2011). Site-specific early season potato yield forecast by neural network in Eastern Canada. Precis. Agric..

[39] Villanueva B.M., Salenga M.L.M. (2018). Bitter Melon Crop Yield Prediction using Machine Learning Algorithm. Int. J. Adv. Comput. Sci. Appl..

[40] Majkovič D., O’Kiely P., Kramberger B., Vračko M., Turk J., Pažek K., Rozman C. (2016). Comparison of using regression modeling and an artificial neural network for herbage dry matter yield forecasting. J. Chemom..

[41] Jeong J.H., Resop J.P., Mueller N.D., Fleisher D.H., Yun K., Butler E.E., Timlin D.J., Shim K., Gerber J.S., Reddy V.R. (2016). Random Forests for Global and Regional Crop Yield Predictions. PLoS ONE.

[42] Hoffman A.L., Kemanian A.R., Forest C.E. (2018). Analysis of climate signals in the crop yield record of sub-Saharan Africa. Glob. Chang. Biol..

[43] Richetti J., Judge J., Boote K.J., Johann J.A., Uribe-Opazo M.A., Becker W.R., Paludo A., Silva L.C.D.A. (2018). Using phenology-based enhanced vegetation index and machine learning for soybean yield estimation in Paraná State, Brazil. J. Appl. Remote Sens..

[44] Wang L., Zhou X., Zhu X., Dong Z., Guo W. (2016). Estimation of biomass in wheat using random forest regression algorithm and remote sensing data. Crop J..

[45] Cunha M., Richter C. (2014). A Time–Frequency Analysis on the Impact of Climate Variability on Semi-Natural Mountain Meadows. IEEE Trans. Geosci. Remote Sens..

[46] Rodrigues A., Marcal A.R.S., Cunha M. (2013). Monitoring Vegetation Dynamics Inferred by Satellite Data Using the PhenoSat Tool. IEEE Trans. Geosci. Remote Sens..

[47] Miao Q., Shi H., Gonçalves J.M., Pereira L.S. (2015). Field assessment of basin irrigation performance and water saving in Hetao, Yellow River basin: Issues to support irrigation systems modernisation. Biosyst. Eng..

[48] Wang Q. (2012). Technical system design and construction of China’s HJ-1 satellites. Int. J. Digit. Earth.

[49] Yu B., Shang S. (2017). Multi-Year Mapping of Maize and Sunflower in Hetao Irrigation District of China with High Spatial and Temporal Resolution Vegetation Index Series. Remote Sens..

[50] Sun C., Liu Y., Zhao S., Zhou M., Yang Y., Li F. (2016). Classification mapping and species identification of salt marshes based on a short-time interval NDVI time-series from HJ-1 optical imagery. Int. J. Appl. Earth Obs. Geoinf..

[51] Pan Z., Huang J., Zhou Q., Wang L., Cheng Y., Zhang H., Blackburn G.A., Yan J., Liu J. (2015). Mapping crop phenology using NDVI time-series derived from HJ-1 A/B data. Int. J. Appl. Earth Obs. Geoinf..

[52] Li X., Zhang Y., Luo J., Jin X., Xu Y., Yang W. (2016). Quantification winter wheat LAI with HJ-1CCD image features over multiple growing seasons. Int. J. Appl. Earth Obs. Geoinf..

[53] Yang Y., Shang S., Jiang L. (2012). Remote sensing temporal and spatial patterns of evapotranspiration and the responses to water management in a large irrigation district of North China. Agric. For. Meteorol..

[54] Jiang L., Shang S., Yang Y., Guan H. (2016). Mapping interannual variability of maize cover in a large irrigation district using a vegetation index-phenological index classifier. Comput. Electron. Agric..

[55] China Center for Resources Satellite Data and Application. http://www.cresda.com.

[56] National Earth System Science Data Sharing Infrastructure. http://spacescience.geodata.cn.

[57] The Bayannur Agricultural Information Network. http://nmyj.bynr.gov.cn.

[58] Allen R.G., Tasumi M., Trezza R. (2007). Satellite-Based Energy Balance for Mapping Evapotranspiration with Internalized Calibration (METRIC)-Model. J. Irrig. Drain. Eng..

[59] Royo C., Aparicio N., Blanco R., Villegas D. (2004). Leaf and green area development of durum wheat genotypes grown under Mediterranean conditions. Eur. J. Agron..

[60] Huang J., Wang H., Dai Q., Han D. (2014). Analysis of NDVI Data for Crop Identification and Yield Estimation. IEEE J. Sel. Top. Appl. Earth Obs. Remote Sens..

[61] Breiman L. (2001). Random Forests. Mach. Learn..

[62] Mutanga O., Adam E., Cho M.A. (2012). High density biomass estimation for wetland vegetation using World View-2 imagery and random forest regression algorithm. Int. J. Appl. Earth Obs. Geoinf..

[63] Vincenzi S., Zucchetta M., Franzoi P., Pellizzato M., Pranovi F., De Leo G.A., Torricelli P. (2011). Application of a Random Forest algorithm to predict spatial distribution of the potential yield of Ruditapes philippinarum in the Venice lagoon, Italy. Ecol. Model..

[64] Peng B., Guan K., Pan M., Li Y. (2018). Benefits of Seasonal Climate Prediction and Satellite Data for Forecasting U.S. Maize Yield. Geophys. Res. Lett..

